# Imaging of a Renal Artery Aneurysm Detected Incidentally on Ultrasonography

**DOI:** 10.1155/2014/375805

**Published:** 2014-05-22

**Authors:** Vasileios Rafailidis, Anna Gavriilidou, Christos Liouliakis, Maria Poultsaki, Triantafyllos Theodoridis, Vasileios Charalampidis

**Affiliations:** ^1^Department of Radiology, General Hospital of Katerini, 6 Km Katerini-Arona, 60100 Katerini, Greece; ^2^Department of Urology, General Hospital of Katerini, 6 Km Katerini-Arona, 60100 Katerini, Greece

## Abstract

Renal artery aneurysms occur with a frequency of less than 1% of the general population. Even if they are usually asymptomatic and incidentally found, they can be complicated with life-threatening conditions like rupture, thrombosis, embolism, or hypertension. Thus, once diagnosed, they should be fully evaluated with further imaging and treated when indicated. We present the case of a patient who was referred for ultrasonography for an unrelated reason. The examination demonstrated a hyperechoic focus near the right kidney. Further imaging workup with MDCT established the diagnosis of a right renal artery aneurysm which was saccular in shape and peripherally calcified. This ring-like calcification was also visible in a KUB radiography which was also performed. After presenting the case, various aspects of this rare entity are discussed.

## 1. Introduction

The term renal artery aneurysm (RAA) characterizes a focal dilatation of the renal artery or its branches. It can be discovered incidentally during workup for hypertension or during abdominal imaging for an unrelated reason (i.e., KUB radiography or ultrasonography) [1]. RAA accounts for 22% of visceral aneurysms [2]. Due to its potential complications, it should be given due consideration with complete imaging, follow-up (MDCT, MRA, and angiography), and treatment when indicated [1]. We present the case of a patient who was diagnosed with an RAA, which was incidentally found on ultrasonography and confirmed with MDCT.

## 2. Case Presentation

A 72-year-old man was referred to the Radiology Department by the Department of Urology for ultrasonography of kidneys, bladder, and prostate for dysuria. He denied history of trauma and his past medical history was free of hypertension or other diseases.

**Figure 1:**
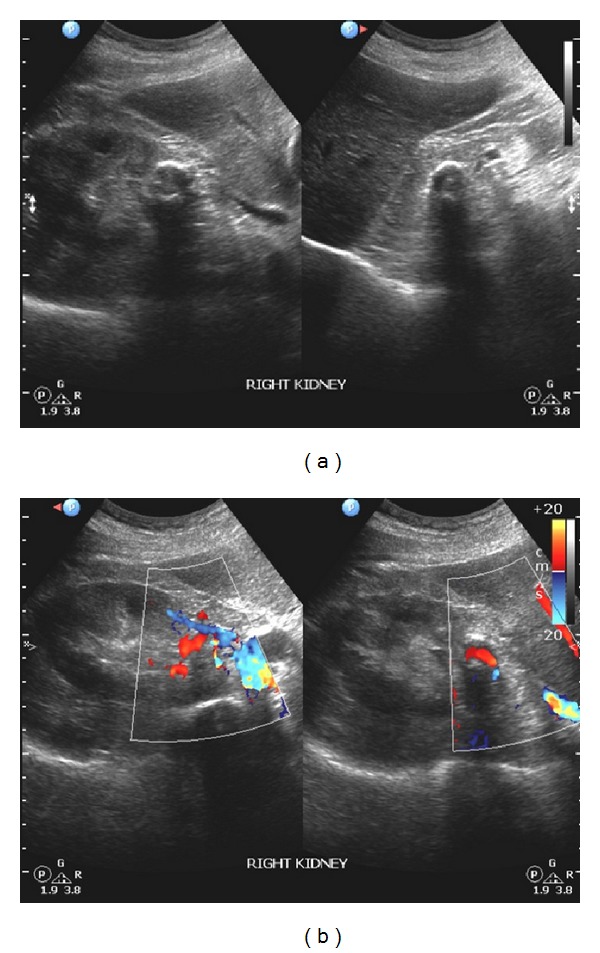
(a) This grey-scale transabdominal ultrasonography image of the right renal area was acquired with a curvilinear multifrequency transducer. It incidentally revealed a curvilinear reflective line with acoustic shadow behind it. This structure was located near the renal pelvis. (b) Color Doppler imaging demonstrated blood flow within the previously described structure. Thus, the structure should be vascular in its nature.

**Figure 2:**
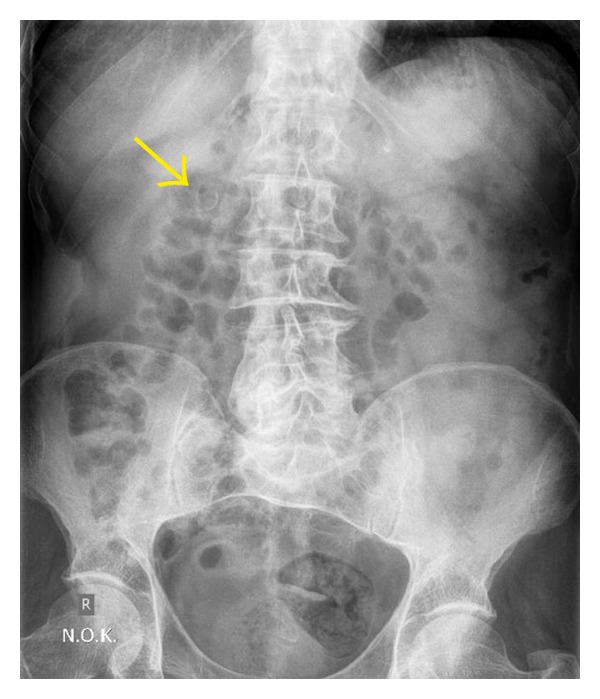
This KUB radiography showed a retroperitoneal curvilinear calcification with discontinuous border, which lied near the right transverse process of the second lumbar vertebra (arrow). This calcification correlated with the ultrasonographically detected pararenal vascular structure with wall calcification.

Ultrasonography revealed that the kidneys were normal except for some cortical cysts of the right kidney. Moreover, there was a hyperechoic focus with acoustic shadow adjacent to the pelvis of the right kidney. These findings were considered to represent a calcified structure of the renal pelvis (Figure 1(a)). At this point, the initial differential diagnosis included a parapelvic cyst, a renal calculus, a tumour, or a renal artery aneurysm. Color Doppler and power Doppler ultrasonography revealed blood flow within this structure, rendering the diagnosis of RAA highly likely (Figure 1(b)). The patient also underwent abdominal radiography which confirmed the presence of a right-sided arc-like, thin calcification with discontinuous border which was located at the level of 2nd lumbar vertebra (Figure 2). This finding was also compatible with a possible RAA.

**Figure 3:**
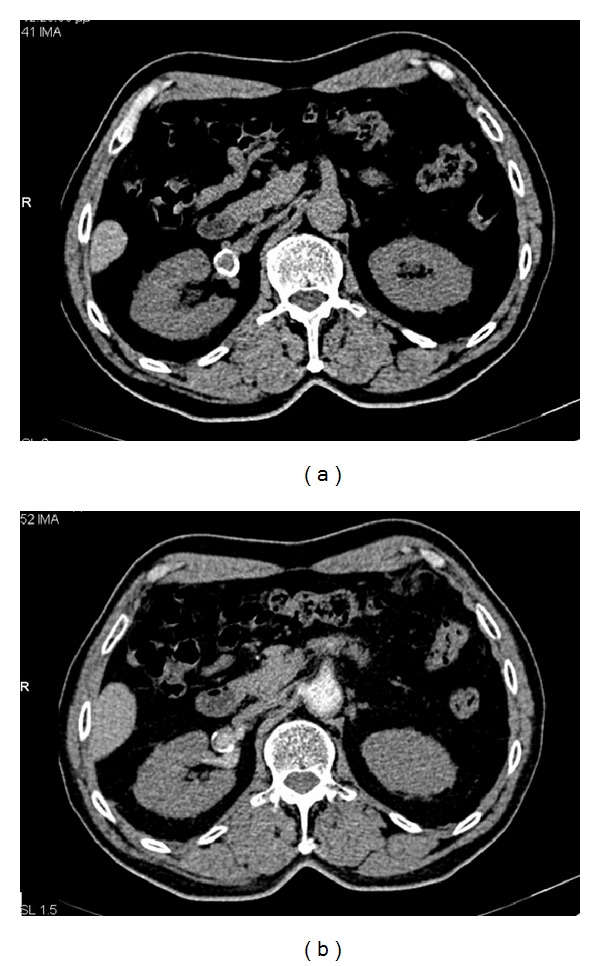
Precontrast (a) and postcontrast (b) axial MDCT images. We can see that the ring-like calcification represented an aneurysm of the right renal artery which had a diameter of 1.8 cm and was saccular in shape.

**Figure 4:**
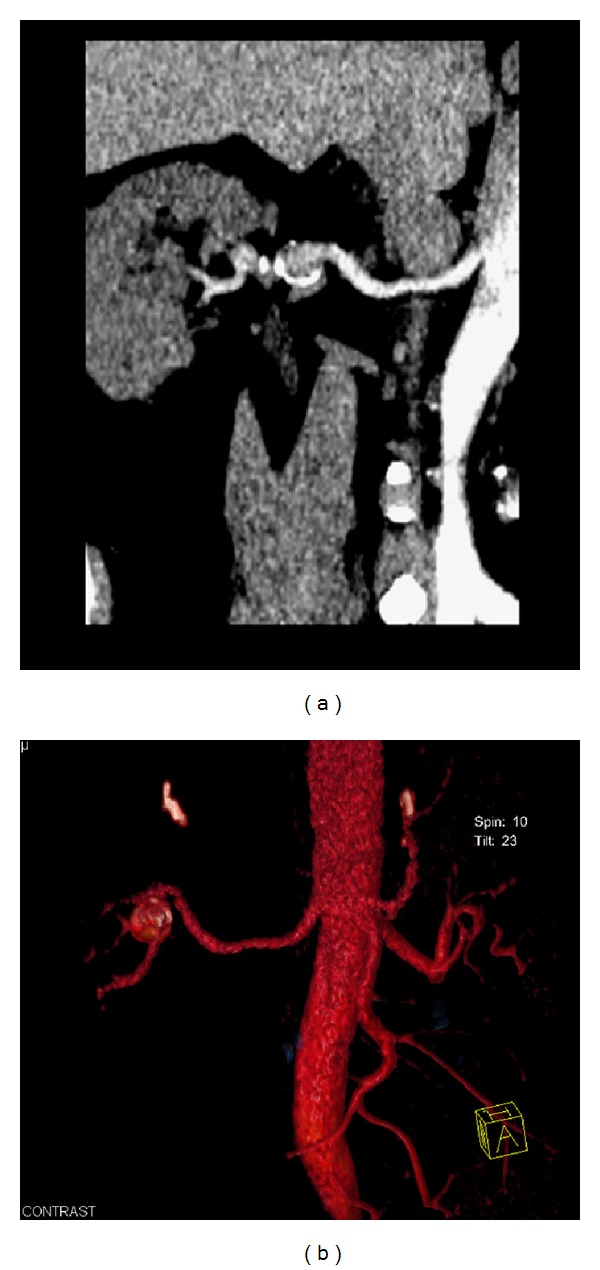
(a) Curved reconstructed image along the right renal artery visualizing in a better way the saccular shape of the aneurysm and the central enhancement from the contrast flow within it. (b) 3D volume rendering technique image of the right renal artery demonstrating the detected aneurysm in an illustrative way and providing us with its detailed anatomy.

Finally, a contrast-enhanced MDCT examination was performed to fully evaluate the suspected RAA and establish the diagnosis. The examination demonstrated the presence of a saccular, peripherally calcified aneurysm of the right renal artery. The aneurysm's diameter was 1.8 cm. After the intravenous administration of contrast medium, the aneurysm showed central enhancement and peripheral thrombus (Figures 3 and 4). After establishing the diagnosis, careful follow-up was chosen as the most appropriate treatment for this patient, who was advised to undergo a digital subtractive angiography or computed tomography angiography one year later to monitor the size of the aneurysm. Up to the present time, the patient had no problem caused by the aneurysm.

## 3. Discussion

The term renal artery aneurysm (RAA) refers to the localized dilatation of the renal artery and/or its branches. The dilatation must be at least twofold in order to be considered an aneurysm [3]. According to a study enrolling adults without renovascular disease, the normal renal artery diameter is approximately 0.5 cm [4]. As it applies in other blood vessels, when this dilatation involves all the layers of the wall of the renal artery, the aneurysm is characterized true. On the other hand, a false aneurysm is created by the surrounding tissues of the vessel. RAA usually affects the right renal artery, as in our case, and has a mean age of diagnosis of 60 years. Its reported prevalence is 0.01% to 1% of the general population. Regarding patients with hypertension, the frequency of the RAA rises to 2.5% and when the hypertension is unresponsive to medical therapy, it can be as high as 39%. In less than 10% of the patients with RAA, the lesion may be located inside the renal parenchyma [1, 2]. In general, there are four types of RAAs: the saccular, fusiform, dissecting, and the arteriovenous/microaneurysm (intrarenal) with the saccular being the most frequent one as it accounts for about 70% of all RAAs. Risk factors for the development of an RAA include renal congenital malformations, untreated hypertension, atherosclerosis, trauma, pregnancy, recent surgery, malignancy, angiomyolipoma of the kidney, radiation exposure, and use of drugs like cyclophosphamide [2].

RAAs usually cause no symptoms but can be complicated by important conditions like rupture, thrombosis, distal embolism, obstructive uropathy, hypertension of renovascular aetiology, and arteriovenous communications [2]. The probability of complications increases as the aneurysm gradually enlarges. Clinical examination of patients with RAA may reveal an ecchymosis and a palpable or pulsatile abdominal mass or bruits [1]. Rupture of an RAA is an important complication which can lead to shock and depends on the age and gender of the patient and the size and histology of the aneurysm. It is characterised by nonspecific symptoms like flank pain and microscopic or gross haematuria but can be suspected when the haematuria worsens with increase of intra-abdominal pressure (e.g., with coughing or defecating). The latter clinical sign can be more frequently encountered in intraparenchymal RAA, a condition named “Wunderlich's syndrome” which requires immediate treatment as it is life-threatening. Rupture of an RAA may also occur in the postpartum period [2, 5].

Imaging is necessary for establishing the diagnosis of an RAA. It has been reported that excretory urography is diagnostic or suggestive of an RAA in only 66% of the patients, whereas angiography poses the diagnosis in 100% of the patients [1]. Frequent excretory urography findings comprise a filling defect or compression of the pyelocalyceal system, a delay in the excretion of the contrast medium, and asymmetry of nephrograms, whereas a rim-like calcification may be the only finding in plain radiography [3].

In general, vascular lesions of the renal sinus like an RAA may initially appear as mass-like lesions. However, their vascular nature is easily identified with color Doppler ultrasonography, contrast-enhanced CT, MRI, and angiography. As more than half of the RAAs have a ring-like calcification, they must be differentiated from renal calculi. This is especially important when extracorporeal shock wave lithotripsy is to be performed [6]. CT and MRA constitute the best noninvasive techniques to assess the vasculature of the kidneys. MRA was shown to be 78% sensitive, 100% specific, and 91% accurate in distinguishing between vascular malformations resembling an aneurysm. When performing a CT or MRA examination of a patient with RAA, the radiologist should evaluate the exact location, size, and structure of the aneurysm as well as its relation to the nearby organs. The degree of contrast enhancement or opacification of an RAA on CT and angiography depends on the amount of thrombus existing within the aneurysm. Spontaneous thrombosis of an RAA was recently reported. MDCT examination identified the vascular origin of the lesion which was not enhanced due to the intraluminal thrombus [7]. Multiplanar and 3D reconstructions of the MDCT examination allow quick and detailed evaluation of the renal arteries and kidneys, permitting exploration of their fine anatomical details. This information is of particular value when planning surgery. Tortuous arteries can be a diagnostic pitfall in axial planes as they falsely resemble aneurysms [1, 3, 6].

As in our case, grey-scale ultrasonography and color Doppler ultrasonography can also raise suspicion of the existence of an RAA. Namely, the calcification of an RAA may be visualized as crescent-shaped echogenic foci with distal acoustic shadowing. The ultrasonographic differential diagnosis of a renal artery aneurysm includes a parapelvic cyst, hydronephrosis, or renal tumours. The usual ultrasonographic appearance of an RAA includes a fluid mass with flow characteristics with Doppler ultrasonography revealing turbulent flow within it. RAA can only rarely cause hydronephrosis as it is usually located in the main renal artery and its primary branches without obstructing the pyelocalyceal system. Finally, nuclear scintigraphy can also be suggestive of this condition [1, 3, 6, 8].

RAA should be treated when causing haemorrhage or uncontrolled hypertension or when they are bigger than 2 to 2.5 cm or they progressively enlarge. Other indications for treatment comprise the presence of an arteriovenous fistula or an RAA greater than 1 cm in women of childbearing age [1]. The RAA can be treated endovascularly by stenting. This procedure is considered to be the treatment of choice and can be performed either electively or in the setting of emergency. Large size and multiplicity are two contraindications of endovascular treatment. In severe cases, open laparotomy and nephrectomy may be needed [1, 2]. Mohan and Stephen reviewed the literature regarding the treatment of peripheral arterial aneurysms on 2013 and concluded that endovascular approach seems to be the best option for RAA [9]. The effectiveness of endovascular treatment was also confirmed in a recent case series where there was 100% technical success rate, no delayed clinical complications, and importantly no major recurrence [10].

## 4. Conclusion

RAA should be always included in the differential diagnosis of parapelvic, pararenal masses with rim-like calcification. Even though they can be asymptomatic and incidentally found, they should always be reported and fully investigated. Furthermore, they should always be followed up and under certain indications treated to avoid life-threatening complications.
